# Quantitative Proteomics to Identify Nuclear RNA-Binding Proteins of Malat1

**DOI:** 10.3390/ijms21031166

**Published:** 2020-02-10

**Authors:** Marian Scherer, Michal Levin, Falk Butter, Marion Scheibe

**Affiliations:** Institute of Molecular Biology (IMB), Ackermannweg 4, 55128 Mainz, Germany; mscher04@students.uni-mainz.de (M.S.); m.levin@imb.de (M.L.); f.butter@imb.de (F.B.)

**Keywords:** quantitative interactomics, lncRNA, RNA-binding proteins, Malat1

## Abstract

The long non-coding RNA Malat1 has been implicated in several human cancers, while the mechanism of action is not completely understood. As RNAs in cells function together with RNA-binding proteins (RBPs), the composition of their RBP complex can shed light on their functionality. We here performed quantitative interactomics of 14 non-overlapping fragments covering the full length of Malat1 to identify possible nuclear interacting proteins. Overall, we identified 35 candidates including 14 already known binders, which are able to interact with Malat1 in the nucleus. Furthermore, the use of fragments along the full-length RNA allowed us to reveal two hotspots for protein binding, one in the 5′-region and one in the 3′-region of Malat1. Our results provide confirmation on previous RNA-protein interaction studies and suggest new candidates for functional investigations.

## 1. Introduction

Mass spectrometry-based proteomics allows identifying thousands of proteins in a single experiment and has been instrumental for screens to identify unknown interaction partners in affinity purification experiments. The first quantitative RNA-protein interaction screens were conducted with in vitro transcribed RNA structures and fragments of mRNA [1,2]. Later, alternative approaches provided catalogues of RNA-binding proteins (RBPs) purified from in vivo cross-linked RNA combined with poly-A capture [3,4] and have been complemented by techniques like capture hybridization analysis of RNA targets and mass spectrometry (CHART-MS), comprehensive identification of RNA-binding proteins by mass spectrometry (ChIRP-MS), and RNA antisense purification and mass spectrometry (RAP-MS) [5,6,7,8] that affinity capture a single target RNA from the in vivo cellular environment using hybridization probes. These and other proteomics strategies have been applied to non-coding RNAs [9].

Metastasis-associated lung adenocarcinoma transcript 1 (Malat1 or MALAT1) or also known as noncoding nuclear enriched abundant transcript 2 (neat2 or NEAT2) was originally discovered as a marker to predict metastasis and survival in early-stage non-small cell lung cancer [10]. MALAT1 deregulation has since then been noted in several human cancers [11,12]. It is a highly transcribed, mostly nuclear transcript with very strong evolutionary conservation in mammals [13], a commonly suggested hallmark of functional importance. The MALAT1 lncRNA precursor transcript in humans has a length of 8779 nt (refseq: NR_002819) and is processed into a full-length lncRNA transcript of ca. 8 kb that localizes to nuclear speckles [14] and a 61 nt mascRNA (MALAT1-associated small cytoplasmic RNA) that functions in the cytoplasm [15]. 

The long noncoding RNA has been involved in splicing, transcriptional, and posttranscriptional regulation [16]. The interaction of lncRNAs with proteins is one possible mechanism for their functionality, for example by acting as a scaffold to bring proteins of different functionality together [17,18]. Thus, several studies using different strategies have been conducted to identify the RBP interactome of MALAT1. From protein-centric studies (mostly CLIP experiments) of known RNA binding proteins, human MALAT1 has been reported to interact with the PRC2 complex acting in transcription repression via methylation of H3K27 [19] as well as with the splicing factor SRSF1 [20,21]. In addition, several RNA-centric approaches have been applied already. A recent SILAC-based RNA interaction screen reported 127 enriched proteins using the 6918–8441 nt fragment of human MALAT1 and unveiled that interaction of DBC1 with this lncRNA does sequester DBC1 from SIRT1 and thereby influences p53 acetylation [22]. Furthermore, human MALAT1 was one target in the recently described multiplexed hybridization purification of RNA-protein complexes for mass spectrometry (HyPR-MS) approach that identified 127 interaction partners to the full-length human transcript [23]. Using ChIRP-MS, another group identified 23 interactors of murine Malat1, including the TEAD proteins whose interaction with Malat1 inhibits their transcriptional activity [24]. While MALAT1 is one of the more highly conserved lncRNAs, overall conservation of lncRNA is not comparable to protein-coding genes. We thus here applied RNA pulldown assays coupled to quantitative mass spectrometry to investigate interaction partners of 14 non-overlapping fragments of murine Malat1 with the aim of (1) investigating whether factors reported previously to be able to bind to human MALAT1 are conserved, (2) focus on the pool of RBPs that are able to interact with Malat1 in the nucleus, and (3) to obtain spatial information of the RBP binding pattern along Malat1.

## 2. Results

### 2.1. Setup of a Quantitative RNA-Protein Interaction Screen for the Long Non-Coding RNA Malat1

**Figure 1:**
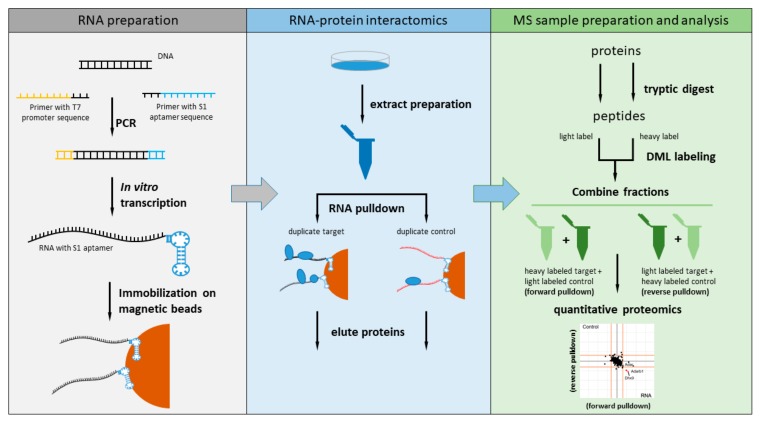
Schematic overview of our quantitative RNA-protein interaction screen of Malat1. RNA fragments of 500 bases length fused with the S1 biotin aptamer at their 3′-end were expressed in vitro, immobilized on paramagnetic streptavidin beads, and then incubated with nuclear enriched lysate. The bound proteins were analyzed by mass spectrometry-based quantitative proteomics.

We decided to measure the RBP interactome for the nuclear 6983 nt murine Malat1 by dividing the lncRNA into 14 fragments of 500 bases each (the last one being 483 nt). Each fragment was amplified by PCR using primers that contained the sequence for the T7 promoter at the 5′-end and the S1 aptamer sequence at the 3′-end (Figure 1). All 14 fragments were in vitro transcribed using T7 RNA polymerase and coupled to paramagnetic streptavidin beads prior to incubation with nuclear extract of the murine NSC-34 neuroblastoma cell line. To perform quantitative comparison against a non-related RNA fragment, we in vitro transcribed a longer version of the region of the pDEST17 vector (469 nt) used as control also in a previous study [2].

The RNA pulldown for each Malat1 fragment and the non-related control RNA was performed twice in parallel to allow incorporation of a label switch during the reductive dimethylation. Unbound proteins were removed by washing and the bound protein fraction eluted with LDS sample buffer. Each pulldown was subjected to tryptic digest and reductive dimethyl labeling prior to mass spectrometry analysis on a high-resolution mass spectrometer.

### 2.2. Quantitative Interactomics of the Malat1 Fragments

**Figure 2:**
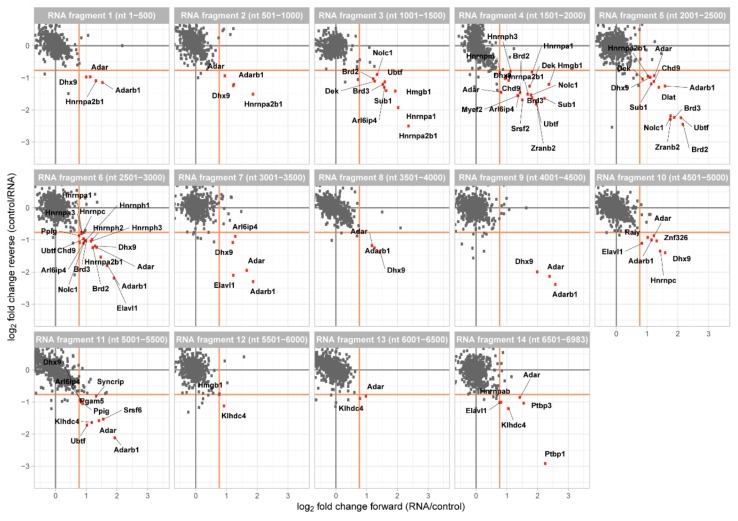
Two-dimensional interaction plot for each of the 14 RNA fragments. Fragments are numbered consecutive from 5′ to 3′ of murine Malat1 and their position within the lncRNA is indicated. Enriched proteins (fold change > 1.7, orange line) are marked in red and annotated.

The data was analyzed with MaxQuant [25] and for each of our Malat1 fragment the quotient of the protein enrichment ratio of the two dimethyl labels was calculated. A forward and reverse experiment were plotted against each other to generate a two-dimensional interaction plot, in which proteins enriched at the Malat1 fragment are shown in the lower right quadrant (Figure 2). Most of the quantified proteins were found to be binding equally well to Malat1 and our control fragment demonstrated by their clustering around the origin of the plot. Notably, across all fragments this background cloud was clearly defined and we thus decided to score RBPs as enriched when their ratio between Malat1 and the control exceeded 1.7-fold (Supplemental Figures S1 and S2).

At this enrichment threshold, we were able to identify enriched proteins at each fragment of Malat1. In agreement with the use of nuclear extracts, 33 of them have been described to localize to the nucleus and 2 were assigned to the mitochondrion. Furthermore, 29 proteins are known to bind to RNA and 20 of them have an annotated RNA recognition domain. Overall, we identified 35 different proteins (Table 1) and the interactors are functionally associated with mRNA processing, RNA splicing, RNA metabolic processes, and gene regulation. These Malat1-enriched interactors include hnRNPs, splicing factors, chromatin remodelers, and RNA editing enzymes.

### 2.3. Binding Pattern of Interactors along Malat1

**Figure 3:**
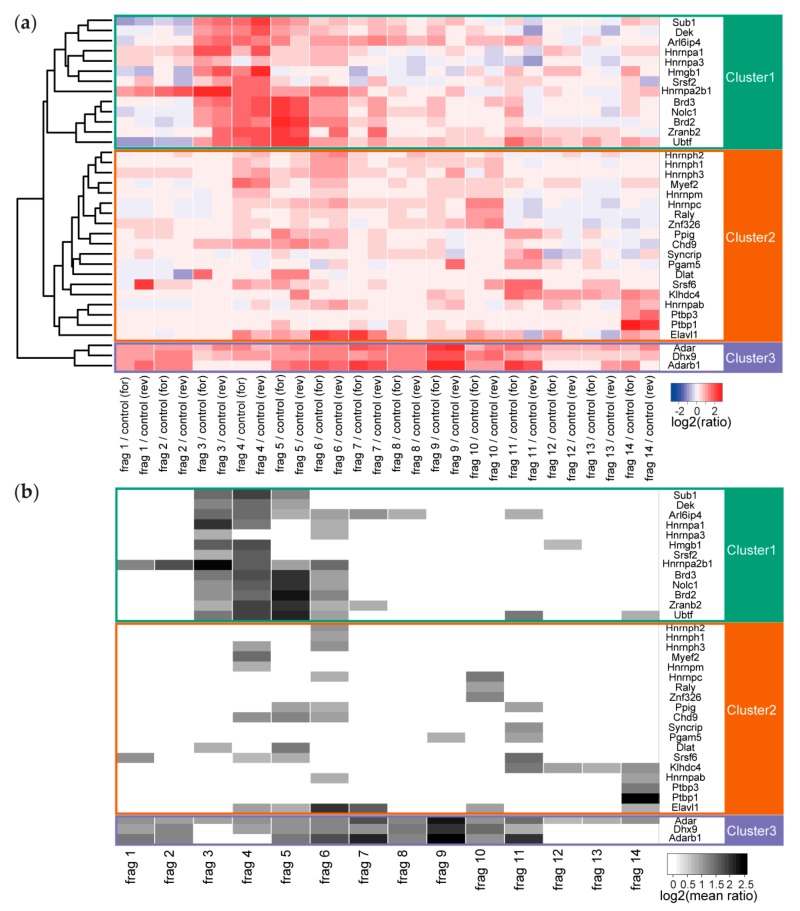
(**a**) Heatmap of all values for the enriched RNA-binding proteins at all Malat1 fragments (including the forward and reverse experiment). Cluster tree on the left edge reveals three major clusters representing different binding patterns of the interactors. (**b**) Filtered heatmap representation of the interactors only when fulfilling the 1.7-fold enrichment threshold.

The binding pattern of the individual proteins ranged from very specific (only enriched with one fragment) to ubiquitous (enriched at all fragments). The number of enriched proteins varied between only 2 enriched interactors for fragment 13 to 20 interactors for fragment 4 and fragment 6. We performed unsupervised clustering of these enriched proteins to investigate whether there is an overall dependency for co-binding and indeed identified 3 major clusters (Figure 3). In the first cluster, we found proteins that are nearly exclusively enriched between fragment 3 and fragment 6, corresponding to 1001–3000 nt of Malat1. In this cluster, we find the heterogeneous RNA binding proteins (Hnrnpa1, Hnrnpa2b1, and Hnrnpa3), proteins involved in chromatin organization (Dek and Hmgb1), transcription (Sub1 and Ubtf), and splicing (Arl6ip4, Brd2, Brd3, Srsf2, and Zranb2). Some proteins are part of complexes, like the spliceosome or have been reported to co-immunoprecipitate in large-scale protein-protein interaction studies; e.g., ARL6IP4/ZRANB2 and BRD2/BRD3 in human HEK293T cells [26].

The second cluster contains all interactors whose binding is distributed across all studied fragments. In addition, here, with Hnrnpab, Hnrnph1, Hnrnph2, Hnrph3, Hnrnpm, Hnrnpc, and Hnrnpq (also known as Syncrip), and the possible group member Raly, the hnRNPs form a large group of interactors. In this group, we also find the splicing factor Srsf6 and the splicing enzyme Ppig, the transcriptional regulators Chd9 and Myef2, the polypyrimidine-tract binding proteins Ptbp1 and Ptbp3, the RNA stability regulator Elavl1 (also known as HuR), the core DBIRD complex member Zfp326 and the uncharacterized kelch domain-containing protein Klhdc4. Two other proteins, Dlat and Pgam5 are reported mitochondrial proteins and while they are detected to be enriched at two different fragments of Malat1, they might not interact with the lncRNA in the nucleus.

In the third cluster, we obtained only 3 proteins (Adar1, Adarb1, and Dhx9). This cluster contains all proteins with broad binding pattern along Malat1, which consequently were enriched on at least 9 of the 14 fragments. These three proteins, one multi-specific RNA helicase and the Adar1/Adarb1 editosome, are nuclear localized and are likely to bind to a large number of cellular RNAs. We might have enriched for these less specific binding proteins through the comparison to an unrelated RNA control sequence that originated from a randomly chosen region of a vector.

### 2.4. Structure of MALAT1

**Figure 4:**
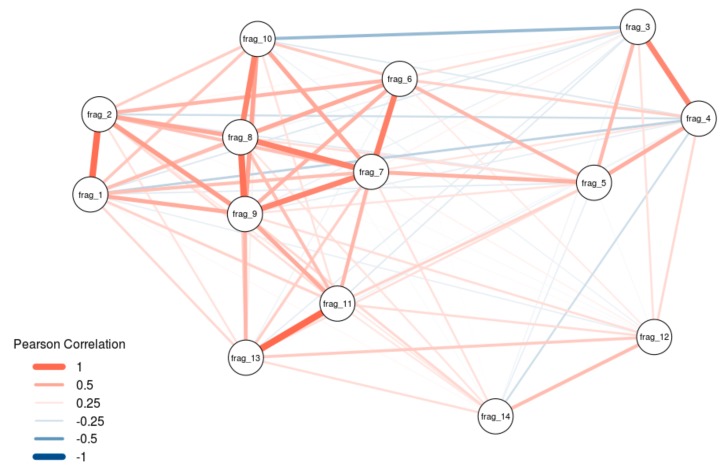
Weighted network diagram describing the resemblance of binding patterns of the different RNA fragments. Nodes represent RNA fragments. Thickness and length of edges connecting nodes correspond to the pairwise Pearson correlation coefficient between the protein enrichments of two RNA fragments. Red and blue colors correspond to positive and negative correlations, respectively.

At least some lncRNAs have been shown to exert their scaffold function by smaller domains as was shown with HOTAIR previously [18]. We thus investigated, whether our detected interactors hint towards functional separated domains in Malat1. Performing network analysis of our 14 fragments, we found that there is a strong correlation among fragments 1–2, 3–4, 6–10, and also fragments 11 and 13, with a slight anti-correlation between parts of the 5′-region (fragment 3–5) and the 3′-region (fragment 6–14) of Malat1 (Figure 4). Notably, the fragments 3–6 (1001–3000 nt) in the 5′-region and the fragments 10–11 (4501–5500 nt) in the 3′-region were identified as hotspots for the binding of proteins.

To note, our analysis shows the highest correlation exclusively with adjacent fragments and anti-correlation is only seen when the fragments are not consecutive. This strongly suggests that the detected protein interaction pattern is not random and shows that Malat1 has regions with similar binding behavior, suggesting possible functional domains.

## 3. Discussion

We performed a quantitative RNA-protein interaction screen using an in vitro RNA pulldown approach for Malat1 with nuclear extract from the murine NSC-34 neuroblastoma cell line. By enrichment analysis, we identified 35 proteins to be able to interact with nuclear Malat1.

A similar quantitative RNA pulldown approach has been performed previously with a fragment of human MALAT1 applying stable isotope labeling of amino acids in cell culture (SILAC) as the quantitative strategy. In contrast to this previous experiment that mapped interactors only at a proposed physiological relevant fragment (nt 6918–8441) of human MALAT1, we here mapped interactors to the complete sequence of mouse Malat1 using 14 fragments of 500 nt each. Additionally, we here used nuclear extract instead of whole cell lysate as the lncRNA Malat1 is localized in the nucleus. While the quantitative proteomic strategies are likely interchangeable, the use of shorter fragments, a cell line originating from a different tissue, a nuclear extract and the study of murine Malat1 are sufficient differences to justify slightly diverse results. Comparing our 35 proteins to the human set of 127 interactors, we found overlap for 10 proteins (Dhx9, Hnrnpa3, Ptbp1, Elavl1, Hnrnpc, Adar, Hnrnpa2b1, Hnrnph1, Hnrnpm, and Hnrnpab), nearly one third of our candidates. Notably, other candidates from the human MALAT1 experiment are cytoplasmic and it is not clear how relevant they are for the function of MALAT1 given its nuclear localization.

Especially noteworthy is that according to conservation analysis the 3′-end of human MALAT1 would be similar to the 5′-end of murine Malat1 [27]. Here again, as in most of the lncRNA world, our understanding of conservation is not as easily applicable as for protein domains. Nevertheless, in general, we find that the hnRNPs involved in (alternative) splicing regulation are able to bind to MALAT1 in both species. In fact, interaction with HNRNPH1 and HNRNPH2 has been shown for human MALAT1 and HNRNPC binding has been mapped to several different fragments of human MALAT1 [28].

In addition to RNA pulldown approaches, oligonucleotide capture experiments have been conducted to obtain RBP interactomes for the complete full-length lncRNA: HyPR-MS for human MALAT1 [23] and ChIRP-MS for murine Malat1 [24]. Our overlap to both experiments is not very high. In the case of the HyPR-MS experiment, we at least overlap for HNRNPH2 and SRSF6; for ChIRP-MS, we have no overlap to the identified 23 interactors. This is not so surprising, as the used techniques are completely different in their experimental strategy and have different limitations. All screens for example, missed the reported interaction of MALAT1 with PRC2 [19].

In our screen, we found candidates that were already functionally associated to either mouse or human MALAT1. This includes the interaction with SRSF2, which has a described functional role in the alternative splicing regulation of MALAT1 [20,29]. Furthermore, we recover the link to the interaction of MALAT1 with DBC1 [22]. While we did not directly enrich DBC1 as an interactor of Malat1, we identified Zfp326 that interacts directly with DBC1 [30,31]. We also detected binding of Elavl1 (also known as HuR) to MALAT1 previously reported to form a complex that represses transcription [32]. Thus, our screen recovered already known interactors, mostly characterized for human MALAT1 also for Malat1 in mouse.

There are some not yet known interactors in our screen that might be interesting candidates for future functional studies. Among the candidates unique to our study are several proteins described to be involved in different cancer types in humans: for example, NOLC1 in multidrug resistant non-small cell lung cancer (the same cancer MALAT1 has originally been identified) [33], DEK in breast cancer [34] and SUB1 in prostate cancer [35]. Intriguingly, we found two bromodomain containing proteins (BRD2 and BRD3) noted to be relevant in cancer [36] interacting with MALAT1. Bromodomains are known to interact with acetylated lysine residues and the second bromodomain of BRD2 is able to bind acetylate histone lysine residues [37], providing a means of Malat1 to be recruited to active genes [6].

One advantage of the RNA pulldown approach is the ability to dissect a long RNA into smaller fragments to identify putative functional domains. We here used 14 fragments of 500 nt length to scan changes in the RBP interactome of Malat1. We identified two hotspots for interaction: fragment 3–6 (nt 1001–3000) and fragment 10–11 (nt 4501–5500). Most of our enriched interaction partners are found at these two regions. This suggests that murine Malat1 might have two distinct domains that provide interaction interfaces for proteins or protein complexes that should be tested functionally in the future.

## 4. Materials and Methods

### 4.1. Cloning of Malat1 from Murine NSC-34 cells

Total RNA was isolated from murine NSC-34 cells using the PrepEase RNA Spin Kit (USB, Cleveland, OH, USA) followed by cDNA preparation with the RevertAid First Strand cDNA Synthesis Kit (Thermo Fisher Scientific, Waltham, MA, USA) according to the manufacturer’s instruction. Malat1 fragments were amplified using Pfu Ultra II Fusion HS DNA polymerase (Agilent, Santa Clara, CA, USA) with specific amplification primers (Supplemental Table S1) and cloned into pCR8 vector using the pCR8/GW/TOPO TA Cloning Kit (Thermo Fisher Scientific, Waltham, MA, USA).

Inserts were validated by Sanger sequencing (Eurofins Genomics, Ebersberg, Germany). The fragment sequences were fused to the T7 promoter sequence at the 5′-end and the minimal S1 aptamer sequence [38] at the 3′-end by a PCR reaction using fragment specific primers (Supplemental Table S2). The control fragment was amplified from a pDEST17 vector (Thermo Fisher Scientific, Waltham, MA, USA) using OneTaq DNA polymerase (NEB, Ipswich, MA, USA), a forward primer containing the T7 promoter sequence and a reverse primer with the minimal S1 aptamer sequence (Supplemental Table S2).

### 4.2. In Vitro Transcription of RNA Fragments

In vitro transcription was performed for 4 h at 37 °C using 200 units of T7 RNA polymerase (Thermo Fisher Scientific, Waltham, MA, USA) in the supplied transcription buffer containing 13 mM NTPs and 1 µg DNA template. Successful transcription was monitored by agarose gel electrophoresis. RNA was purified by phenol-chloroform extraction. To this end, the transcription reaction was combined with nuclease free water to a final volume of 250 µL. Then, 400 µL Phenol/Chloroform 5:1 pH 4.3–4.7 (Sigma Aldrich, St. Louis, MO, USA) was added and the mixture was vortexed. After centrifugation for 3 min at 10,000 rpm (Fresco centrifuge, Thermo, Waltham, MA, USA), the upper phase was transferred into a new reaction tube and 1 mL pure ethanol (Sigma Aldrich, St. Louis, MO, USA) containing 15 µg/mL GlycoBlue (Ambion, Austin, TX, USA) was added and mixed. The sample was incubated for 10 min at −80 °C and centrifuged afterwards for 1 h at 4 °C and 14,000 rpm (Fresco centrifuge, Thermo Fisher Scientific, Waltham, MA, USA). The RNA pellet was dried for 5 min at room temperature and resuspended in nuclease free water. RNA concentration was determined by measuring the extinction at 260 nm with a Nanodrop 2000 spectral photometer (Thermo Fisher Scientific, Waltham, MA, USA).

### 4.3. Preparation of Nuclear Extracts

NSC-34 cells were grown in DMEM media (Thermo Fisher Scientific, Waltham, MA, USA) containing 10% FBS (Thermo Fisher Scientific, Waltham, MA, USA) and 100 U/mL penicillin and 100 μg/mL streptomycin (Sigma Aldrich, St. Louis, MO, USA) in a humidified incubator at 37 °C, 5% CO_2_, and 70% humidity. Nuclear extracts were prepared basically as described previously [39]. In short, harvested cells were washed in 1x PBS (Thermo Fisher Scientific, Waltham, MA, USA), incubated in five pellet volumes of cold buffer A (10 mM HEPES–KOH pH 7.9, 1.5 mM MgCl_2_, 10 mM KCl) for 10 min on ice and collected by centrifugation. Cells were resuspended in two volumes of cold buffer A containing 0.1% IGEPAL CA-630 (Sigma Aldrich, St. Louis, MO, USA), 0.5 mM DTT, and a protease inhibitor mix (1 µM Pepstatin A [SERVA Electrophoresis GmbH, Heidelberg, Germany], 1 µg/mL Leupeptin [SERVA Electrophoresis GmbH, Heidelberg, Germany], and 1 mM PMSF [SERVA Electrophoresis GmbH, Heidelberg, Germany]) and sheared with 50 strokes (type B pestle) in a dounce homogenizer. After centrifugation for 15 min at 3900 rpm (Multifuge X3R, Thermo Fisher Scientific, Waltham, MA, USA) at 4 °C, pelleted nuclei were resuspended in two volumes of buffer C (420 mM NaCl, 20 mM HEPES–KOH pH 7.9, 20% glycerol, 2 mM MgCl_2_, 0.2 mM EDTA pH 8.0, 0.1% IGEPAL CA-630, 0.5 mM DTT, and protease inhibitor mix). Subsequently the suspension was incubated for 1 h on a rotating wheel at 4 °C and centrifuged at 14,500 rpm (Fresco centrifuge, Thermo Fisher Scientific, Waltham, MA, USA) for 1 h at 4 °C to obtain the nuclear extract as supernatant. The protein concentration was established by Bradford (Bio-rad, Hercules, CA, USA) using an UV/Vis photometer (Amersham Bioscience, Freiburg, Germany).

### 4.4. RNA Pulldown

For each pulldown, 25 µg S1-tagged RNA was immobilized on 50 µL paramagnetic streptavidin beads (Dynabeads C1, Thermo Fisher Scientific, Waltham, MA, USA) in RNA binding buffer (100 mM NaCl, 50 mM HEPES–KOH pH 7.6, 0.1% IGEPAL CA-630, 10 mM MgCl_2_, 1 µM Pepstatin A, 1 µg/mL Leupeptin, and 1 mM PMSF) at 4 °C for 30 min on a rotation wheel. After two washing steps with RNA binding buffer to remove excess unbound RNA, the prepared beads were incubated with 400 µg nuclear extract and 20 µg yeast tRNA (Thermo Fisher Scientific, Waltham, MA, USA) as competitor in RNA wash buffer (250 mM NaCl, 50 mM HEPES–KOH pH 7.6, 0.1% IGEPAL CA-630, 10 mM MgCl_2_, 1 µM Pepstatin A, 1 µg/mL Leupeptin, and 1 mM PMSF) for 30 min and 4 °C on a rotation wheel. Unbound proteins were removed by three washing steps with 200 µL RNA washing buffer and bound proteins eluted by heating beads in 1× LDS Buffer (Thermo Fisher Scientific, Waltham, MA, USA) supplemented with 100 mM DTT for 10 min at 70 °C and 1400 rpm in a thermomixer (Eppendorf, Hamburg, Germany).

### 4.5. Mass Spectrometry Sample Preparation and Dimethyl Labeling

Protein samples were separated on a 4%–12% NuPAGE Novex Bis-Tris precast gel (Thermo Fisher Scientific, Waltham, MA, USA) for 8 min at 180 V in 1× MES buffer (Thermo Fisher Scientific, Waltham, MA, USA). After protein fixation and coomassie blue staining for protein detection, gels (one slice per sample) were mince and destained in 50% EtOH/25 mM ammonium bicarbonate (ABC) followed by dehydration in 100% acetonitrile (ACN). Afterwards a reduction reaction was performed by incubating samples for 1 h in 10 mM DTT, 50 mM ABC at 56 °C followed by an alkylation reaction in alkylation buffer (50 mM iodoacetamide (Sigma Aldrich, St. Louis, MO, USA) in 50 mM ABC) for 45 min at room temperature in the dark. Gel slices were washed with 50 mM TEAB buffer pH 8.0 and dehydrated again with ACN. Dried gel pieces were subsequently incubated over night with trypsin solution (1 μg trypsin [Sigma Aldrich, St. Louis, MO, USA]) in 50mM TEAB per sample) at 37 °C. Tryptic peptides were extracted twice with 30% ACN and three times with pure ACN. The mixture with the extracted peptides was concentrated in a speed-vac (Eppendorf, Hamburg, Germany) to a final volume of ca. 100 µL. Dimethyl labeling was performed as described [40,41]. Peptides were either incubated with formaldehyde (Sigma Aldrich, St. Louis, MO, USA) and NaBH_3_CN (Sigma Aldrich, St. Louis, MO, USA) leading to a 28 Da mass-tag (light labeled fraction) or with formaldehyde-d2 (Sigma Aldrich, St. Louis, MO, USA) and NaBH_3_CN resulting in a 32 Da mass-tag (heavy labeled fraction). Heavy labeled peptides from pulldowns with Malat1 fragments were mixed with light-labeled peptides from the control RNA pulldown (forward experiment) and vice versa (reverse experiment).

### 4.6. Mass Spectrometry Measurement and Data Analysis

Peptides were desalted on StageTips [42] and separated on a capillary (New Objective, Woburn, MA, USA) packed with Reprosil C18 (Dr. Maisch GmbH, Ammerbuch-Entringen, Germany). The column was attached to an Easy nLC 1000 system (Thermo Fisher Scientific, San Jose, California, USA) operated with a gradient from 5% to 60% acetonitrile in 0.1% formic acid at a flow of 225 nL/min. The spray capillary was mounted on the nanospray ion source of a Q Exactive Plus mass spectrometer (Thermo Fisher Scientific, Bremen, Germany). Measurements were using HCD fragmentation with a data-dependent Top10 MS/MS spectra acquisition scheme per MS full scan in the Orbitrap analyzer. The mass spectrometry proteomics data have been deposited to the ProteomeXchange Consortium via the PRIDE [43] partner repository with the dataset identifier PXD017309. The raw files were processed with MaxQuant (version 1.5.2.8) and searched against the mouse UniProt database (54,220 entries). MaxQuant standard settings were used, except dimethyl labels (Lys0, Nter0, Lys4, and Nter4) were defined.

### 4.7. Bioinformatics Analysis

Contaminants, reverse database hits, protein groups only identified by site, and protein groups with less than 2 peptides (at least one of them classified as unique) were removed by filtering from the proteinGroups file. Missing values were imputed by shifting a compressed normal distribution obtained from the LFQ intensity values to the limit of quantitation. The two-dimensional interactions plots were generated from the filtered MaxQuant proteinGroups output file using in-house R scripts [44]. The quantitative data for Figure 2 (zoom) and Supplementary Figure S1 (full) can be retrieved from Supplementary Table S1. Protein localization information were obtained from human orthologues at UniProt [45] or ProteinAtlas [46]. Cluster analysis was performed using the stats package in R (version 3.4.4). Network analysis and illustration (Figure 4) were performed using the qgraph package in R (version 3.4.4). Figure 3 is based on data provided in Supplementary Table S2.

## Figures and Tables

**Table 1:** List of the proteins found to be enriched at murine Malat1. Proteins marked in bold were already known to interact with human or mouse MALAT1 from previous studies. Proteins with a gene ontology annotation for RNA-binding are shaded in grey.

Protein	Name/Function	Primary Localization
**Adar1**	**dsRNA specific RNA deaminase**	**Nuclear**
Adarb1	dsRNA specific editase	Nuclear
Arl6ip4	modulating alternative pre-mRNA splicing	Nuclear
Brd2	binds hyperacetylated histones, regulation of transcription	Nuclear
Brd3	binds hyperacetylated histones, regulation of transcription	Nuclear
Chd9	transcriptional coactivator and putative chromatin remodeler	Nuclear
Dek	chromatin organization	Nuclear
**Dhx9**	**multifunctional RNA helicase**	**Nuclear and cytoskeleton**
Dlat	dihydrolipoamid S-acetyltransferase	Mitochondrial
**Elavl1**	**HuR antigen, binds and stabilizes mRNA**	**Nuclear**
**Hmgb1**	**high mobility group protein B1**	**Nuclear and endosome**
Hnrnpa1	heterogeneous nuclear ribonucleoprotein A1	Nuclear
**Hnrnpa2b1**	**heterogeneous nuclear ribonucleoprotein A2B1**	**Nuclear and extracellular**
**Hnrnpa3**	**heterogeneous nuclear ribonucleoprotein A3**	**Nuclear**
**Hnrnpab**	**heterogeneous nuclear ribonucleoprotein AB**	**Nuclear**
**Hnrnpc**	**heterogeneous nuclear ribonucleoprotein C**	**Nuclear**
**Hnrnph1**	**heterogeneous nuclear ribonucleoprotein H1**	**Nuclear**
**Hnrnph2**	**heterogeneous nuclear ribonucleoprotein H2**	**Nuclear**
Hnrnph3	heterogeneous nuclear ribonucleoprotein H3	Nuclear
**Hnrnpm**	**heterogeneous nuclear ribonucleoprotein M**	**Nuclear**
Klhdc4	kelch domain-containing protein 4	Nuclear
Myef2	transcriptional repressor of the myelin basic protein gene	Nuclear
Nolc1	regulator of RNA polymerase I	Nuclear
Pgam5	serine/threonine phosphatase	Mitochondrial
Ppig	peptidyl-prolyl cis-trans isomerase G, pre-mRNA splicing	Nuclear
**Ptbp1**	**pre-mRNA splicing and regulation of alternative splicing**	**Nuclear**
Ptbp3	pre-mRNA alternative splicing	Nuclear
Raly	possible heterogeneous nuclear ribonucleoprotein	Nuclear
**Srsf2**	**serine/arginine-rich splicing factor 2**	**Nuclear**
**Srsf6**	**serine/arginine-rich splicing factor 6**	**Nuclear**
Sub1	coactivator that functions cooperatively with TAFs	Nuclear
Syncrip	heterogeneous nuclear ribonucleoprotein Q	Nuclear and ER
Ubtf	nucleolar transcription factor binding rRNA gene promoter	Nuclear
Zfp326	core component of DBIRD complex, alternative splicing	Nuclear
Zranb2	splice factor required for alternative splicing	Nuclear

## Supplementary Materials

Supplementary materials can be found at https://www.mdpi.com/1422-0067/21/3/1166/s1.

## Abbreviations

CHART-MS	capture hybridization analysis of RNA targets and mass spectrometry
ChIRP-MS	comprehensive identification of RNA-binding proteins by mass spectrometry
ER	endoplasmic reticulum
HyPR-MS	multiplexed hybridization purification of RNA-protein complexes for mass spectrometry
lncRNA	long non-coding RNA
MS	mass spectrometry
RAP-MS	RNA antisense purification and mass spectrometry
RBP	RNA-binding protein
SILAC	stable isotope labeling of amino acids in cell culture
