# Supplementary figures and images for: Quantitative Proteomics to Identify Nuclear RNA-Binding Proteins of Malat1
Source: Int J Mol Sci. 2020 Feb 10;21(3):1166. doi: 10.3390/ijms21031166 (PMC7037011; doi:10.3390/ijms21031166)

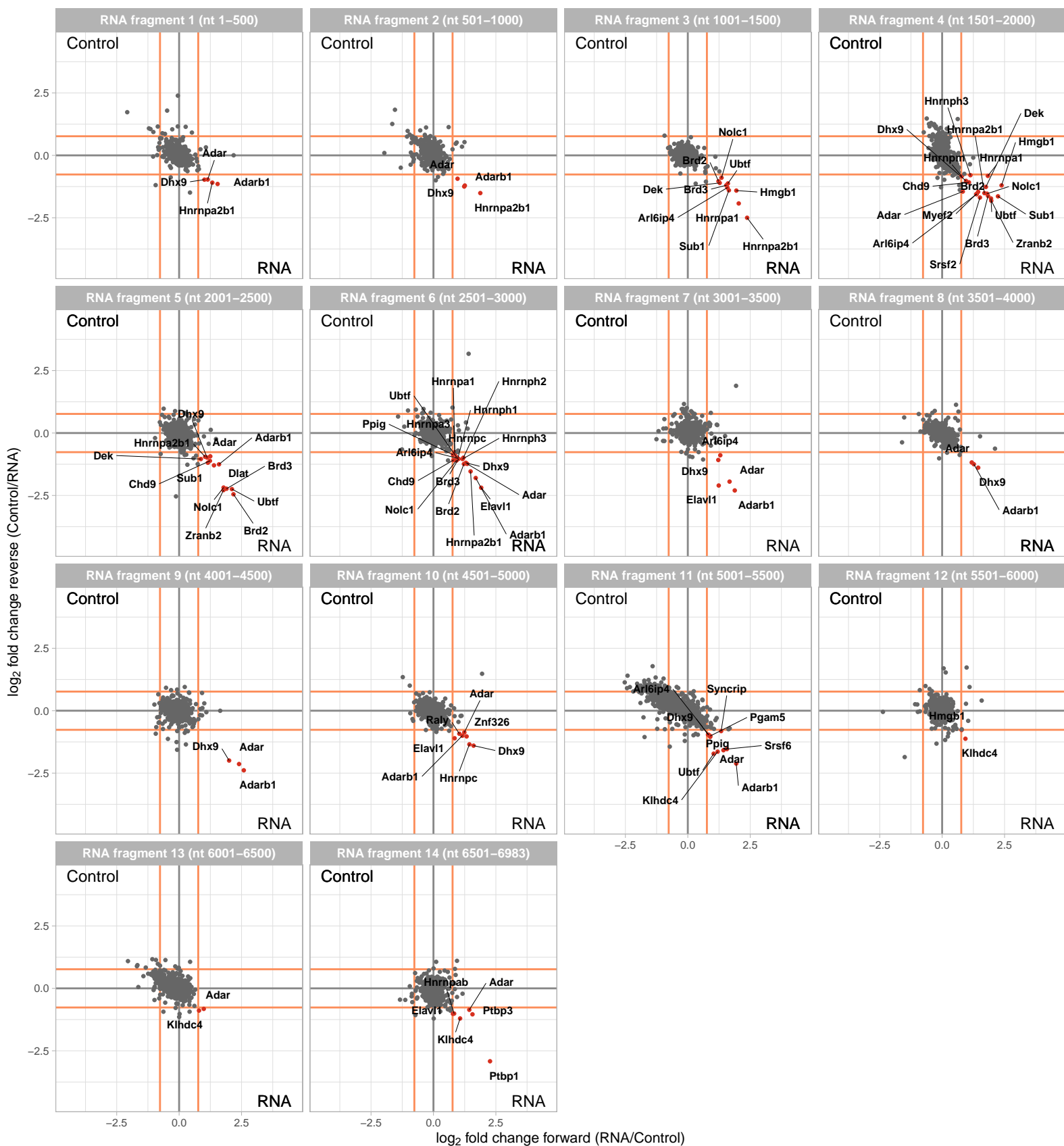

Supplement: Supplementary file 1 [file ijms-21-01166-s001.zip › SupplFigure1.pdf]
